# A Randomized Phase II Study of S-1 Adjuvant Chemotherapy With or Without Hochu-ekki-to, a Japanese Herbal Medicine, for Stage II/III Gastric Cancer: The KUGC07 (SHOT) Trial

**DOI:** 10.3389/fonc.2019.00294

**Published:** 2019-04-17

**Authors:** Hiroshi Okabe, Yousuke Kinjo, Kazutaka Obama, Hisahiro Hosogi, Hiroaki Hata, Yoshito Asao, Hideki Harada, Dai Manaka, Atsushi Itami, Satoshi Teramukai, Yoshiharu Sakai

**Affiliations:** ^1^Department of Surgery, Graduate School of Medicine, Kyoto University, Kyoto, Japan; ^2^Department of Gastroenterological Surgery, New Tokyo Hospital, Matsudo, Japan; ^3^Department of Surgery, Himeji Medical Center, Himeji, Japan; ^4^Department of Surgery, Kyoto City Hospital, Kyoto, Japan; ^5^Department of Surgery, Osaka Red Cross Hospital, Osaka, Japan; ^6^Department of Surgery, Kyoto Medical Center, Kyoto, Japan; ^7^Department of Abdominal Surgery, Tenri Hospital, Nara, Japan; ^8^Department of Surgery, Hirakata Kohsai Hospital, Osaka, Japan; ^9^Department of Surgery, Shiga General Hospital, Moriyama, Japan; ^10^Department of Surgery, Katsura Hospital, Kyoto, Japan; ^11^Department of Surgery, Kobe City Nishi-Kobe Medical Center, Kobe, Japan; ^12^Department of Biostatistics, Graduate School of Medical Science, Kyoto Prefectural University of Medicine, Kyoto, Japan

**Keywords:** gastric cancer, adjuvant chemotherapy, S-1, herbal medicine, Hochu-ekki-to

## Abstract

**Objectives:** A multicenter randomized phase II study was conducted to evaluate the effects of Hochu-ekki-to (TJ-41) for reducing adverse reactions and increasing compliance with S-1 adjuvant therapy for advanced gastric cancer.

**Methods:** The eligibility criteria were pathological stage II/III after R0 resection. Patients received adjuvant therapy with S-1 alone (group S) or S-1 with TJ-41 (group ST) for 1 year. The primary endpoint was the completion rate of S-1. Secondary endpoints were adverse events, relative dose intensity, relapse-free survival (RFS), and overall survival (OS).

**Results:** We randomly assigned 56 patients to group ST and 57 patients to group S. The completion rates of S-1 were 54.5 and 50.9%, the median relative dose intensities were 89.2 and 71.9%, and adverse events of grade 3 or 4 occurred in 45.5 and 54.5% in groups ST and S, respectively. There was no significant difference in 3-year OS or RFS between the two groups.

**Conclusions:** TJ-41 does not increase relative dose and completion rate of S-1 significantly. J-41 may reduce toxic effects, but our findings do not support routine use of TJ-41 after gastrectomy.

## Introduction

The standard treatment for advanced gastric cancer is surgical resection with D2 lymphadenectomy followed by adjuvant chemotherapy in Eastern countries, where gastric cancer is common, while perioperative chemotherapy or chemoradiation is preferred in Western countries (1–3). Adjuvant chemotherapy with S-1 for 1 year yields a 5-year survival rate of 72%, reducing the risk of death by 33% compared to surgery alone (4). However, compliance with adjuvant chemotherapy following radical gastrectomy is unsatisfactory. The ACTS-GC trial found that 35% of patients drop out during a 1-year course of S-1 administration (1). Completion rates of S-1 for 1 year in other prospective studies were even lower, from 39 to 63% (5–7). Furthermore, even for patients who took S-1 for 12 months, the dose was decreased in 47% of cases. Adverse events are the most common reason for discontinuation or dose reduction, including anorexia, nausea, diarrhea, stomatitis, and hematological events. Both early termination of treatment and a decrease of relative dose intensity are closely related to lower survival; therefore, increasing compliance by reduction of toxicity is likely to improve survival of these patients (8).

Hochu-ekki-to (TJ-41, Chinese name: Bu-zhong-yi-qi-tang, Korean name: Bojungikki-tang) is a traditional herbal medicine that originated in China and is composed of 10 species of medicinal plant. It has long been used for general fatigue, weakness, and loss of appetite (9). Several reports have shown biological effects of TJ-41 augmenting immune responses, reducing surgical stress, and preventing side effects of chemotherapy (9–11). TJ-41 is often prescribed to cancer patients in Japan, and has beneficial effects on cancer-related fatigue and quality of life in these patients (12). We conducted a multicenter randomized phase II study to test our hypothesis that TJ-41 may improve compliance with S-1 by reducing its adverse reactions, and improve survival of patients with advanced gastric cancer.

## Patients and Methods

### Eligibility Criteria

The eligibility criteria included histologically proven gastric cancer of pathological stage II/III after R0 resection according to the 14th Japanese classification of gastric carcinoma, except for T3N0 (13); negative peritoneal washing cytology, without liver metastasis; age 20–80 years old; S-1 started within 8 weeks after surgery; provision of written informed consent; and adequate organ function (white blood cells (WBC) ≥3,000 to ≤12,000 /mm^3^, neutrophils ≥1,500 /mm^3^, hemoglobin ≥9 g/dL, platelets ≥100,000 /mm^3^, total bilirubin ≤1.5 mg/dL, aspartate aminotransferase (AST) ≤100 IU/L, alanine aminotransferase (ALT) ≤ 100 IU/L, creatinine clearance calculated by the Cockcroft-Gault formula ≥60 mL/min). Patients were excluded if they had synchronous cancer; preoperative chemotherapy or radiotherapy; severe comorbidities such as bowel obstruction, uncontrolled diabetes, severe diarrhea, pulmonary fibrosis or heart disease; a requirement for a H2 blocker, proton pump inhibitor, warfarin, phenytoin, or flucytosine; or a contraindication for S-1.

### Study Design and Sample Size

The primary endpoint was the completion rate of adjuvant chemotherapy with S-1 for 1 year. Secondary endpoints were adverse events of adjuvant chemotherapy, relative dose intensity, relapse free survival (RFS), and overall survival (OS). A total sample size of 110 patients was determined to have 80% power at a one-sided alpha of 0.2 for detecting 15% improvement of the baseline completion rate of 65%. Relative dose intensity was defined as the ratio of actual dose of S-1 administered to the planned dose in 1 year. Patients were considered to have completed adjuvant chemotherapy when they received S-1 for 1 year at a relative dose intensity of ≥70%.

Patients were randomly assigned to the S-1 plus TJ-41 (ST) group or the S-1 alone (S) group with a centralized computer system stratified by cancer stage (II or III), institutions, and type of surgery (total gastrectomy or other type of resection) at an outsourced data center. Patients were to be followed for 3 years. OS was defined as the time between the date of registration and the date of death. RFS was defined as the time between the date of registration and the date of relapse or death, whichever came first. Relapse was determined by clinical signs or imaging such as computed tomography (CT), endoscopy, or gastrointestinal radiography. Evaluations by CT and based on CEA and CA19-9 levels were performed at least every 6 months for the first 3 years. Data for event-free patients were censored on the date the patient was last seen. The protocol was first approved by the ethics review committee of Kyoto University on January 2011, and then approved by all participating centers. The original accrual period was planned to be 2 years, with an additional 3 years of follow-up, but was extended by 5 months due to delayed accrual. This trial is registered at the UMIN Clinical Trials Registry (UMIN000004701).

### Adjuvant Chemotherapy and Adverse Events

Adjuvant chemotherapy was initiated within 8 weeks after surgery. In group S, patients received oral S-1 at 80 mg/m^2^/day for 4 weeks, followed by a 2-week recovery period. Patients with body surface areas <1.25 m^2^, 1.25 to <1.5 m^2^, and ≥1.5 m^2^ received 80, 100, and 120 mg of S-1 daily, respectively. This 6-week cycle was repeated for 1 year after surgery. In group ST, 7.5 g/day of TJ-41 was additionally administered every day during the same period.

Adverse events were assessed using the Common Toxicity Criteria for Adverse Events (CTCAE, version 4.0). S-1 was interrupted during the course when patients had hematologic toxicity of grade 3, or, 4, or non-hematologic toxicity of grade 2 or above, including appetite loss, nausea, vomit, stomatitis, diarrhea, impaired hearing, and neurological or cardiac disorders. Initiation of the next course was postponed until recovery and fulfillment of the following criteria: WBC ≥2,000 /mm^3^, neutrophils ≥1,000 /mm^3^, platelets ≥75,000 /mm^3^, total bilirubin ≤3 mg/dL, AST/ALT ≤150 IU/L, and creatinine clearance ≥30 mL/min. The daily dose of S-1 was reduced from 120 mg to 100 mg, 100 mg to 80 mg, and 80 mg to 50 mg when the following adverse events occurred in the previous course: leukocytopenia or neutropenia of grade 4, other toxic effect of grade 3 or 4, and creatinine clearance <60 ml/min. When the association of adverse events and S-1 was unclear, or adverse events occurred only after 14 days of S-1 administration, modification to a 3-week cycle (S-1 on days 1-14 of each cycle followed by a 1-week recovery period) was allowed, instead of dose reduction. When adverse events of grade 3 or above that were associated with TJ-41 occurred, TJ-41 was discontinued.

The protocol treatment was discontinued when patients withdrew the consent, recurrences were diagnosed, severe adverse events preventing treatment occurred, general condition deteriorated severely, comorbidities deteriorated severely, intended protocol violation was noticed, or according to physicians' discretion.

### Statistical Analysis

For patient characteristics and efficacy endpoints, continuous variables were compared by Wilcoxon rank sum test and categorical variables were compared by chi-squared test. The frequency of adverse events between treatment groups was tested using Fisher's exact test. Time-to-event endpoints were analyzed by the Kaplan-Meier method and compared using a stratified log-rank test with clinical stage and type of surgery as stratification factors. Cox proportional hazards model was used to estimate hazard ratios and their 95% confidence intervals. To examine if TJ-41 had a beneficial effect on completion of adjuvant therapy, subgroup analyses of completion rates in both groups were analyzed based on age, sex, pathological stage, and type of surgery. To estimate of odds ratios and to test for treatment group by each factor interaction, logistic regression analysis was used. The significant level was one-sided 0.2 for the primary endpoint, a value of 0.05 was considered to indicate statistical significance in the other analyses. All the analyses were conducted with SAS version 9.4, JMP 13 (SAS Institute, Cary, NC, USA) and SPSS Statistics version 19.0 (IBM, Armonk, NY, USA).

## Results

### Characteristics of the Patients

**Figure 1:**
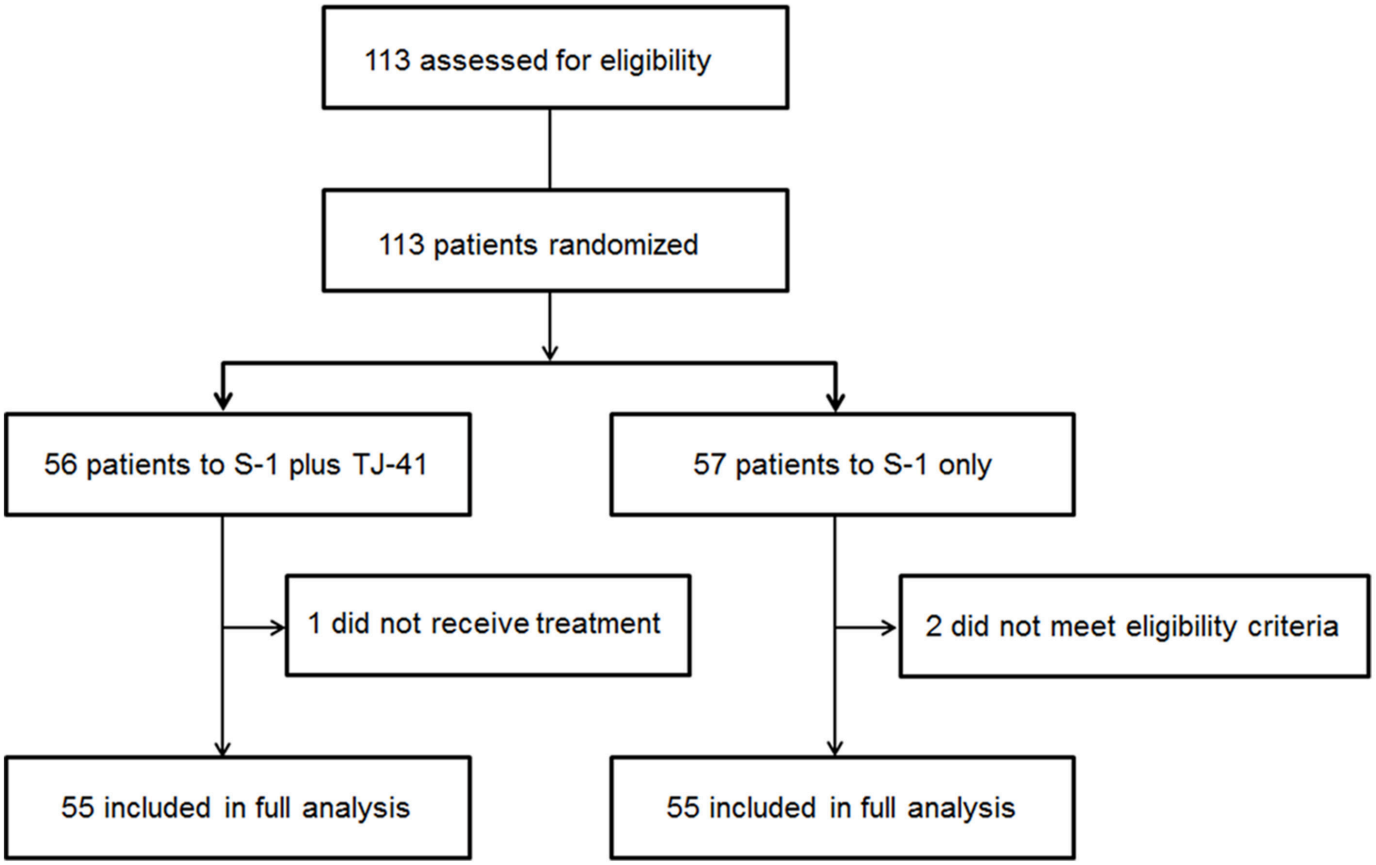
Flow diagram of the 113 registered patients. Two patients treated with S-1 alone (group S) were diagnosed as T3N0, which did not meet the eligibility criteria.

Between March 2011 and July 2013, 113 patients from 25 hospitals were enrolled and assigned to treatment with S-1 plus TJ-41 (group ST, *n* = 56) or S-1 alone (group S, *n* = 57). After randomization, three patients were found to be ineligible due to pathological T3N0 (*n* = 2 in group S) and not receiving study medication (*n* = 1 in group ST). Therefore, the main analyses were based on data from 110 eligible patients (*n* = 55 in each group, Figure 1). The baseline characteristics of the 110 patients were similar in the two groups (Table 1).

**Table 1:** Background characteristics of the patients.

**Characteristic**	**S-1 plus TJ-41**	**S-1 only**	P^*^
	**(*N* = 55)**	**(*N* = 55)**	
Sex			1.000
Male	37 (67.3)	37 (67.3)	
Female	18 (32.7)	18 (32.7)	
Age (median, range)	65, 49 to 76	64, 35 to 77	0.322
Neoadjuvant Chemotherapy			0.185
No	48 (87.3)	52 (94.5)	
Yes	7 (12.7)	3 (5.5)	
Type of Surgery			0.699
Distal/proximal gastrectomy	33 (60.0)	31 (58.4)	
Total gastrectomy	22 (40.0)	24 (43.6)	
T Category			0.088
T1	3 (5.5)	1 (1.8)	
T2	9 (16.4)	16 (29.1)	
T3	13 (23.6)	19 (34.5)	
T4	30 (54.6)	19 (34.5)	
N Category			0.281
N0	8 (14.5)	6 (10.9)	
N1	13 (23.6)	19 (34.5)	
N2	13 (23.6)	17 (30.9)	
N3	21 (38.2)	13 (23.6)	
Stage			0.561
II	21 (38.2)	24 (43.6)	
III	34 (61.8)	31 (56.4)	

*Data are shown as number (%), except for age*.

* *P-value for age calculated by Wilcoxon rank sum test. P-values for other factors calculated by chi-squared test*.

### Completion of Adjuvant Chemotherapy

Adjuvant chemotherapy with S-1 for 1 year was completed by 30 patients (54.5%, 95%CI: 41.4–67.7%) in group ST and 28 (50.9%, 95%CI: 37.7–64.1%) in group S, with no significant difference in the completion rate between the two groups (one-sided *P* = 0.35). Cycle interruptions were needed in 24 (43.6%) and 34 (61.8%) patients, the S-1 dose was reduced in 17 (30.9%) and 21 (38.2%) patients, and cycle delay occurred in 13 (23.6%) and 14 (25.5%) patients in groups ST and S, respectively. TJ-41 was discontinued in two patients (3.6%). Reasons of discontinuation were bad taste and appetite loss. No significant interaction between the treatment groups and any of these variables was found in S-1 completion rate (**Figure 3**).

### Relative Dose Intensity and Adverse Events

The median relative dose intensity of S-1 was 89.2% in group ST and 71.9% in group S (*P* = 0.33). The difference was not significant, but the percentage of patients who achieved a relative dose intensity of 90% was marginally greater in group ST [26 (47.3%) vs. 17 (30.9%), *P* = 0.08]. Adverse events were the most common reason for discontinuation of S-1 in both groups (ST vs. S: 36 vs. 48%), followed by recurrence (36 vs. 30%), patients' request (12 vs. 15%), and miscellaneous (16 vs. 7%). Reasons and time points of discontinuation in each group were shown in Table 2. During the treatment period, 25 patients (45.5%) in group ST and 30 (54.5%) in group S had adverse events of grade 3 or above (*P* = 0.446), with no significant difference in the frequency and severity of adverse events between the groups (Table 3).

**Table 2:** Reasons and time points of discontinuation of S-1.

**Reasons of discontinuation of S-1**	**Time points of discontinuation of S-1**			
	**-3 months**	**4-6 months**	**7-12 months**	**Total**
**S-1 PLUS TJ-41**				
Adverse events	8	0	1	9
Recurrences	0	2	7	9
Patients' request	2	0	1	3
Miscellaneous	3	1	0	4
Total	13	3	9	25
**S-1 ONLY**				
Adverse events	6	3	4	13
Recurrences	1	4	3	8
Patients' request	1	0	3	4
Miscellaneous	2	0	0	2
Total	10	7	10	27

**Table 3:** Adverse events in the two treatment groups at different CTCAE grades.

**Adverse event**	**S-1 plus TJ-41**		**S-1 only**		***P***-value^*^	
	**(*****N*** **=** **55)**		**(*****N*** **=** **55)**			
	**Grade ≥1**	**Grade ≥3**	**Grade ≥1**	**Grade ≥3**	**Grade ≥1**	**Grade ≥3**
**HEMATOLOGIC**						
Leukocytopenia	30 (54.5)	2 (3.6)	30 (60.0)	3 (5.5)	0.700	1.000
Neutrocytopenia	31 (56.4)	8 (14.5)	33 (60.0)	11 (20.0)	0.847	0.615
Anemia	48 (87.3)	5 (9.1)	50 (90.9)	0 (0)	0.761	0.057
Thrombocytopenia	22 (40.0)	1 (1.8)	23 (41.8)	2 (3.6)	1.000	1.000
Total bilirubin	34 (61.8)	0 (0)	28 (50.9)	1 (1.8)	0.336	1.000
Aspartate aminotransferase	18 (32.7)	2 (3.6)	21 (38.2)	1 (1.8)	0.690	1.000
Alanine aminotransferase	21 (38.2)	1 (1.8)	21 (38.2)	1 (1.8)	1.000	1.000
Creatinine	4 (7.3)	0 (0)	5 (9.1) 0	0 (0)	1.000	1.000
**NON-HEMATOLOGIC**						
Nausea	26 (47.3)	1 (1.8)	28 (50.9)	1 (1.8)	0.849	1.000
Vomiting	8 (14.5)	1 (1.8)	16 (29.1)	1 (1.8)	0.105	1.000
Diarrhea	21 (38.2)	2 (3.6)	28 (50.9)	6 (10.9)	0.250	0.271
Appetite loss	33 (60.0)	2 (3.6)	36 (65.5)	4 (7.3)	0.694	0.679
Stomatitis	12 (21.8)	1 (1.8)	15 (27.3)	3 (5.5)	0.658	0.618
General malaise	25 (45.5)	1 (1.8)	27 (49.1)	4 (7.3)	0.849	0.363
Skin rash	17 (30.9)	1 (1.8)	10 (18.2)	1 (1.8)	0.183	1.000
Pigmentation	18 (32.7)	NA	15 (27.3)	NA	0.678	NA
Dysgeusia	11 (20.0)	NA	13 (23.6)	NA	0.818	NA
Total	54 (98.2)	25 (45.5)	55 (100)	30 (54.5)	1.000	0.446

*CTCAE, Common Toxicity Criteria of Adverse Events of the National Cancer Institute (version 4.0); NA, not applicable*.

* *P-values calculated by Fisher's exact test*.

### Survival

**Figure 2:**
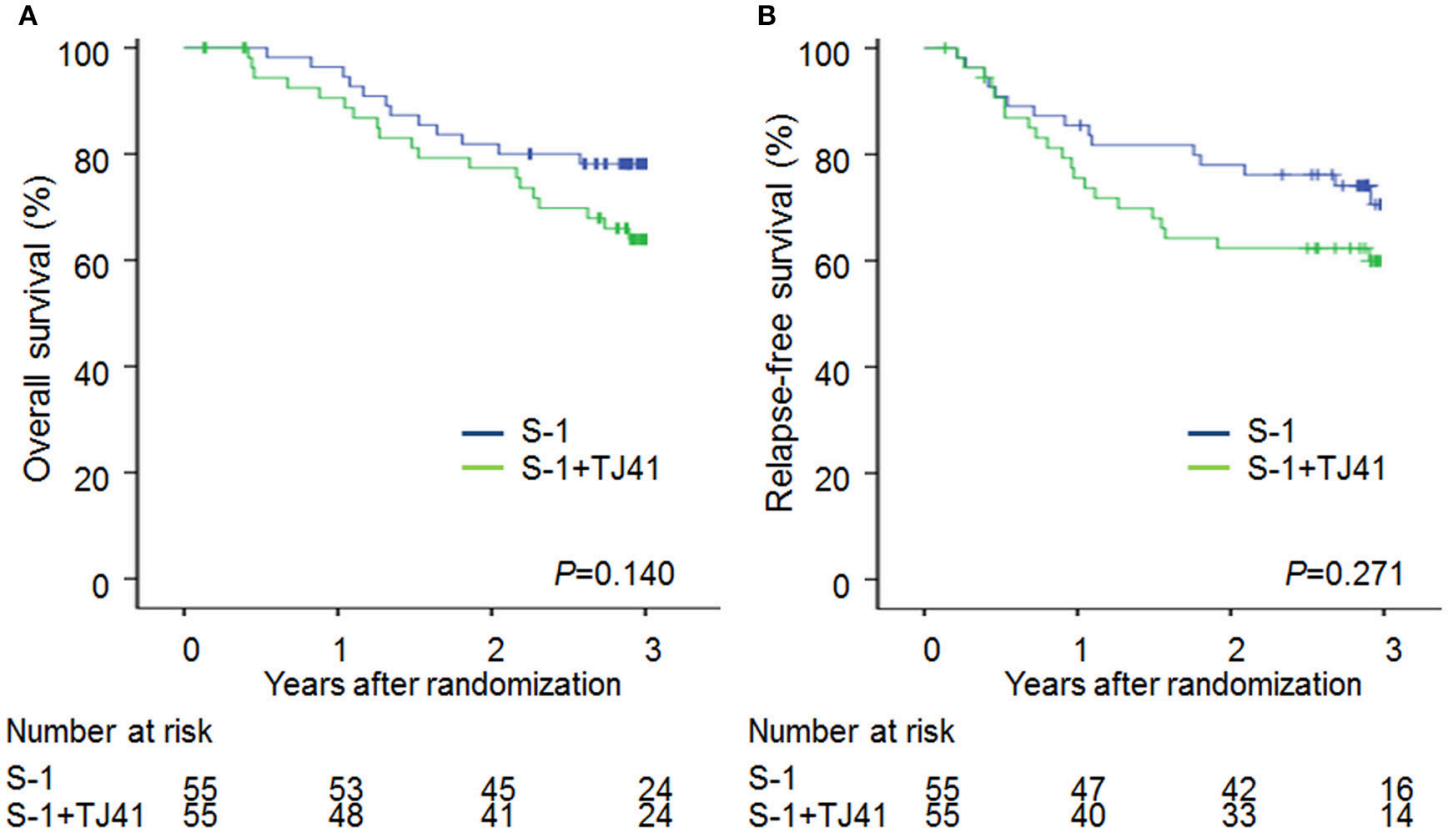
Overall survival **(A)** and relapse-free survival **(B)** curves in patients treated with S-1 alone (group S) and S-1 plus TJ-41 (group ST).

The median follow-up period was 1,076 (range, 49–1751) days in group ST and 1,084 (range, 207–1801) days in group S. The 3-year OS rates were 63.9% (95% CI, 50.2–75.7) and 78.1% (95% CI, 65.3–87.1) (Figure 2A) and the 3-year RFS rates were 59.9% (95% CI, 46.2–72.3) and 70.6% (95% CI, 56.2–81.8) (Figure 2B) in groups ST and S. The hazard ratio for death and relapse in the group ST compared with S was 1.68 (95% CI, 0.84–3.48, *P* = 0.140), and 1.44 (95% CI, 0.75–2.81, RFS (*P* = 0.271), respectively.

## Discussion

The primary aim of this study was to evaluate if addition of Hochu-ekki-to (TJ-41) increases compliance with S-1 after D2 gastrectomy for advanced gastric cancer. We found no statistical difference of 1-year completion rate of S-1 between the two groups. Therefore, these results do not support routine use of TJ-41 after gastrectomy for advanced gastric cancer.

However, there were marginal differences between the two groups in relative dose intensity (89 vs. 72%) and rate of grade 3 or 4 adverse events (46 vs. 55%). Most of the adverse events except for jaundice and skin rash were decreased with the use of TJ-41. Although jaundice and skin rash in some patients might be caused by TJ-41, there was no apparent severe side effects of TJ-41. These findings suggest that TJ-41 does have some positive effects in reducing toxicity, but the study was underpowered to detect those effects. We calculated the sample size based on a hypothesis that TJ-41 would increase the completion rate from 65 to 80%. In hindsight, this expectation was too high based only on decreasing adverse effects of S-1, because 15% of patients discontinued S-1 because of recurrence. This recurrence rate was consistent with the ACTS-GC study based on a careful evaluation of the RFS curves (1).

**Figure 3:**
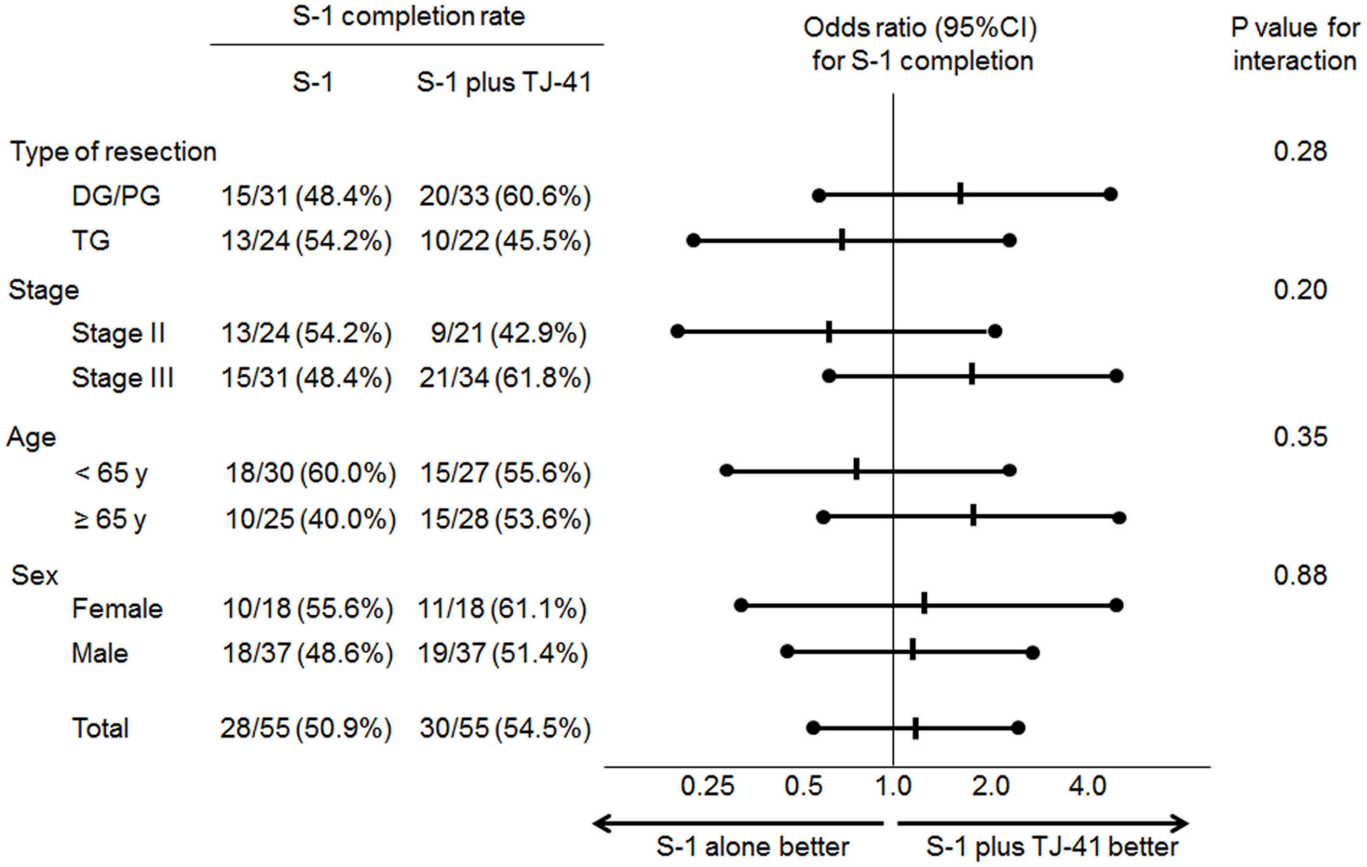
Odds ratios (ORs) for completion of S-1 adjuvant therapy and *P*-values for the interaction of treatment groups and clinical factors.

Although completion rate of S-1 was not different between patients with or without TJ-41, subgroup analyses showed that the completion rate for S-1 tends to be higher in elderly patients (≥ 65 years) who received TJ-41 (Figure 3). TJ-41 is usually prescribed for elderly patients with severe weakness, and several functions including improvement of cancer-related fatigue, increased oral food intake, and enhancement of immunological responses were reported (12, 14). Therefore, optional use of TJ-41 may be reasonable in elderly patients after gastrectomy. However, to clearly identify target patients who could benefit from TJ-41 following gastrectomy, further study is needed.

The overall and relapse-free survival was slightly better in patients with S-1 only, while the completion rate of S-1 was slightly higher in patients with S-1 plus TJ-41. Although the difference was not significant, this was contrary to our expectation that better completion rate would lead to better survival. It was difficult to fully explain this unexpected result, but higher proportion of T4 or N3 in patients with S-1 plus TJ-41 may be related to more frequent recurrence.

One limitation of this study is that the design was not double-blinded. Because TJ-41 is an herbal medicine that has a unique scent, developing a placebo is difficult and would require considerable time and cost. We planned to conduct this explorative phase II study first before conducting a double-blind randomized controlled trial. Another limitation is that we did not screen patients from the point of view of traditional Chinese medicine. Because Chinese prescription is supposed to work for patients in a certain status, selection of patients would be necessary to evaluate its efficacy properly.

Current recommended adjuvant chemotherapy regimens following gastrectomy differ somewhat among countries. These regimens include S-1; capecitabine and oxaliplatin (CapeOX); and epirubicin, oxaliplatin, and oxaliplatin (EOX) (2, 4, 15). The efficacy of each has been shown in clinical trials, but low compliance rates are a common problem for these regimens. Although this was a negative study, to find new measures to increase compliance with current regimens, as well as development of new agents to clear residual cancer cells, would be an important strategy to improve survival of patients after gastrectomy for gastric cancer.

## Ethics Statement

This study was carried out in accordance with the recommendations of Ethical Guidelines for Medical and Health Research Involving Human Subjects, Ministry of Health, Labor and Welfare, with written informed consent from all subjects. All subjects gave written informed consent in accordance with the Declaration of Helsinki. The protocol was approved by Kyoto University Graduate School and Faculty of Medicine Kyoto University Hospital Ethics Committee.

## Author Contributions

HO, YK, and KO designed the study. ST and HO analyzed the data. HO drafted the manuscript. KO, HisH, HirH, YA, HidH, DM, and AI collected and registered the data. HO, KO, and YS interpreted the data, and revised the manuscript. All authors read and approved the final manuscript.

### Conflict of Interest Statement

YS received research grant from TSUMURA & CO. The remaining authors declare that the research was conducted in the absence of any commercial or financial relationships that could be construed as a potential conflict of interest.
